# Alterations of brainstem volume in patients with first-episode and recurrent major depressive disorder

**DOI:** 10.1186/s12888-023-05146-4

**Published:** 2023-09-21

**Authors:** Yue Chen, Lili Jia, Weijia Gao, Congchong Wu, Qingli Mu, Zhe Fang, Shaohua Hu, Manli Huang, Peng Zhang, Shaojia Lu

**Affiliations:** 1 Department of Psychiatry, The First Affiliated Hospital, Zhejiang University School of Medicine, Key Laboratory of Mental Disorder’s Management of Zhejiang Province, Zhejiang Engineering Center for Mathematical Mental Health, No. 79 Qingchun Road, Hangzhou, Zhejiang 310003 China; 2 Faculty of Clinical Medicine, Zhejiang University School of Medicine, Hangzhou, Zhejiang China; 3 Department of Clinical Psychology, The Fifth Peoples’ Hospital of Lin’an District, Hangzhou, Zhejiang China; 4 Department of Child Psychology, The Children’s Hospital, National Clinical Research Center for Child Health, Zhejiang University School of Medicine, National Children’s Regional Medical Center, Hangzhou, Zhejiang China; 5 Department of Psychiatry, Affiliated Xiaoshan Hospital, Hangzhou Normal University, No. 728 North Yucai Road, Hangzhou, Zhejiang 311200 China

**Keywords:** Major depressive disorder, First-episode, Recurrence, Brainstem, Superior cerebellar peduncle

## Abstract

**Background:**

Major depressive disorder (MDD) is a prevalent mental health condition characterized by recurrent episodes in a substantial proportion of patients. The number of previous episodes is one of the most crucial predictors of depression recurrence. However, the underlying neural mechanisms remain unclear. To date, there have been limited neuroimaging studies investigating morphological changes of the brainstem in patients with first-episode MDD (FMDD) and recurrent MDD (RMDD). This study aimed to examine volumetric changes of individual brainstem regions in relation to the number of previous episodes and disease duration.

**Method:**

A total of 111 individuals including 36 FMDD, 25 RMDD, and 50 healthy controls (HCs) underwent T1-weighted structural magnetic resonance imaging scans. A Bayesian segmentation algorithm was used to analyze the volume of each brainstem region, including the medulla oblongata, pons, midbrain, and superior cerebellar peduncle (SCP), as well as the whole brainstem volume. Analyses of variance (ANOVA) were performed to obtain brain regions with significant differences among three groups and then *post hoc* tests were calculated for inter-group comparisons. Partial correlation analyses were further conducted to identify associations between regional volumes and clinical features.

**Results:**

The ANOVA revealed significant brainstem volumetric differences among three groups in the pons, midbrain, SCP, and the whole brainstem (*F* = 3.996 ~ 5.886, adjusted *p* = 0.015 ~ 0.028). As compared with HCs, both groups of MDD patients showed decreased volumes in the pons as well as the entire brainstem (*p* = 0.002 ~ 0.034), however, only the FMDD group demonstrated a significantly reduced volume in the midbrain (*p* = 0.003). Specifically, the RMDD group exhibited significantly decreased SCP volume when comparing to both FMDD (*p* = 0.021) group and HCs (*p* = 0.008). Correlation analyses revealed that the SCP volumes were negatively associated with the number of depressive episodes (*r*=-0.36, *p* < 0.01) and illness duration (*r*=-0.28, *p* = 0.035) in patients with MDD.

**Conclusion:**

The present findings provided evidence of decreased brainstem volume involving in the pathophysiology of MDD, particularly, volumetric reduction in the SCP might represent a neurobiological marker for RMDD. Further research is needed to confirm our observations and deepen our understanding of the neural mechanisms underlying depression recurrence.

## Introduction

Major depressive disorder (MDD) is a serious and prevalent mental disorder that has become one of the leading contributors to the global burden of disease and disability [1]. MDD is characterized by a highly variable course and inconsistent response to treatment [2]. Recurrent episodes of depression after remission become a common feature of MDD and the number of episodes has been demonstrated to be one of the best predictors for the risk of recurrence [3, 4]. Previously, a considerable amount of research has been conducted to investigate the potential mechanisms of MDD, however, the neurobiological alterations underlying depression recurrence remain unclear.

Over the past few decades, neuroimaging has emerged as an important approach to discover brain structural and functional alterations and subsequently has been used to investigate the pathophysiology of MDD since its origin. Although not consistent, previous studies performed on patients with single-episode MDD and multiple-episode MDD have identified a number of depression relapse-associated neural signatures [5–9]. For example, volumetric differences in the amygdala, prefrontal cortex (PFC), and habenula have been found in patients with recurrent MDD compared with first-episode patients [10–12]. The occurrence of depressive episodes has been specifically linked to gray matter volume (GMV) changes in the insula, hippocampus, and amygdala, as well as cortical thickness decline in the dorsomedial PFC (dmPFC) [13–15]. Moreover, disease duration as a clearly distinguishable marker of MDD recurrence has also been proved to be negatively associated with GMV in the dmPFC, insula, hippocampus, subgenual anterior cingulate cortex (sgACC), thalamus, and nucleus accumbens (NAc) [16–18]. Taken together, these findings clearly point toward adverse effects of disease progression on the morphology of reported brain regions, however, the brainstem, a crucial neural node in the neurobiology of MDD, has not been specifically investigated in patients with first-episode and recurrent MDD.

The brainstem is believed to play a significant role in the pathophysiology of MDD. Disturbances of monoaminergic neurotransmitters and the hypothalamic-pituitary-adrenal (HPA) axis are proved to be involved in the biological mechanisms of MDD [19]. The HPA axis is regulated by multiple neural systems, including those present in the brainstem [20, 21]. Key nuclei in the midbrain and pons, such as the nucleus raphes dorsalis (DRN), locus coeruleus, and ventral tegmental area (VTA), are responsible for the majority of monoamine neurotransmitter synthesis [22]. The DRN, situated in the brainstem, has been identified as having a close relationship with MDD [23]. These neurochemical transmitter disorders of the monoaminergic system are considered to be at the root of the underlying pathophysiology of MDD [24].

Brainstem is composed of the medulla oblongata, pons and midbrain, having the function of regulation of the cardiac, respiratory, and central nervous systems including consciousness and the sleep cycle [25]. Different sub-regions of brainstem have different connections and functions. In MDD patients, it was reported that reward-related learning deficits was associated with striatal-midbrain connectivity [26]. Moreover, a recent meta-analysis revealed a marginally significant cluster of altered intrinsic activity was found between patients with treatment-resistant depression (TRD) and health controls in the cerebellum and pons [27]. However, morphologic changes of the brainstem and the sub-regions including oblongata, pons, midbrain and superior cerebellar peduncle (SCP), in MDD have rarely been reported in neuroimaging studies. About 3 decades ago, in an ultrasound study, it was reported that structural disintegration of the brainstem raphe was observed in patients with MDD [28]. In recent years, the midbrain has been found to be enlarged in patients with a current depressive episode [29, 30], and interestingly, this volume may return to normal after antidepressant treatment, and is even reduced, when the patient is in remission [30]. It has also been found that MDD patients exhibited an increased white matter volume in the superior brainstem tegument, a region containing several nuclei which are associated with the pathologic hypothesis of MDD [13]. Therefore, exploring the volume changes of brainstem subregions can provide greater spatial sensitivity and a wider understanding of pathophysiological basis of MDD.

Some studies of the brainstem volume in MDD have produced contradictory results. A prior study which used voxel-based morphometry (VBM) analysis reported decreased gray matter concentrations in areas around the DRN in 47 patients with MDD [16]. Meanwhile, a recent study revealed that female patients with MDD showed non-significant volumetric differences in the subcortical regions, whole brainstem, and each brainstem region compared to healthy controls [31]. It is worth noting that the current findings are always inconsistent and various factors may be associated with brain structural changes in patients with MDD, for instance, gender, illness duration, severity of symptoms, or times of depressive episodes.

In this context, the aims of the present study were to investigate volume changes in each region of the brainstem in patients with first-episode and recurrent MDD, and specifically to evaluate the impact of recurrence on brainstem volume in MDD patients. Based on previous findings, we hypothesized that morphologic changes of the brainstem could be involved in the pathophysiology of MDD, and depressive recurrence might lead to distinctive deficits of brainstem substructures in MDD patients.

## Methods

### Participants

In this study, 61 patients diagnosed with MDD, including 36 patients with first-episode MDD (FMDD) and 25 patients with recurrent-episode MDD (RMDD), were recruited from Department of Psychiatry, The First Affiliated Hospital, Zhejiang University School of Medicine. All subjects satisfied the Diagnostic and Statistical Manual of Mental Disorders, IV Edition (DSM-IV) criteria for MDD and met the following criteria: (1) aged 18–45 years; (2) drug-naïve patients with first-episode depression or recurrent depression with continued withdrawal of more than 3 months; (3) total score of the 17-item Hamilton Depression Scale (HAMD-17) [32] ≥ 17; and (4) right-handedness. Meanwhile, a total of 50 age- and sex-matched healthy controls (HCs) were recruited from local residents, hospital staffs and students. All HCs were thoroughly interviewed and were free from any current or lifetime history of psychiatric disorders according to the DSM-IV criteria. According to the number of depressive episodes, MDD patients were grouped into two groups: first episode depressed individuals (FMDD group) and recurrent-episode depressed individuals (RMDD group). Patients with FMDD met the criteria that the total number of depressive episodes (including current episode) = 1, duration of current depressive episode is over than 2 weeks and no history of drug treatment. And patients with FMDD met the criteria that the total number of depressive episodes (including current episode) > 1, duration of current depressive episode is over than 2 weeks and withdrawal time over three months. The general exclusion criteria for all subjects were as follows: (1) existence of any major medical disease including cardiovascular, respiratory, endocrine and neurological diseases (e.g., epilepsy, brain trauma, and stroke); (2) current use of any medication that might affect the central nervous system, (3) drug or alcohol dependence or abuse; (4) female with pregnancy; (5) with histories of psychotherapy and physical therapy, such as transcranial direct current stimulation (tDCS), transcranial magnetic stimulation (TMS), and electroconvulsive therapy (ECT); 6) contraindications to MRI scan, including retractors or braces, metallic implants, and claustrophobia. The present study is one of our serial investigations focusing on MDD and the recruitment of participants has been described in our previous studies [33–35]. This study was approved by the local Medical Ethics Committee of The First Affiliated Hospital, Zhejiang University School of Medicine. Prior to commencement of the study, all participants provided written informed consent.

### Clinical assessments

Clinical assessments were conducted by two highly qualified psychiatrists. The demographic and clinical data including age, sex, years of education, medical history (previous history of depression, disease duration, number of episodes and onset age) and medication use was collected by a self-designed questionnaire from all the participants. The Structured Clinical Interview for DSM-IV (SCID) consisting of 11 modules is a standard interview for evaluating psychiatric diagnoses. It was used in this study for the diagnostic assessment of MDD and further psychiatric disorders, which was also administered to each subject. The severity of depression was evaluated by using HAMD-17 [32], which has been demonstrated to have a good interrater reliability and internal reliability. It is the most commonly used clinician rating of depressive symptom severity and higher scores indicated more severe depression.

### MRI acquisition

Imaging data were acquired on a 3.0-T scanner (Signa, HDxt, GE healthcare, USA) with a standard birdcage head coil in the Magnetic Resonance Center belonging to The First Affiliated Hospital, Zhejiang University School of Medicine. All participants were instructed to lie still with their eyes closed and to avoid falling asleep. The protocol in this study involved Sagittal 3D T1-weighted structural images, which were acquired by a brain volume (BRAVO) sequence with the following parameters: TR = Minimum (7.3 ms), TE = Minimum (3.0 ms), TI = 1100 ms, flip angle = 7, FOV = 256 * 256 mm^2^, Matrix = 256 * 256, slice thickness = 1 mm, bandwidth = 31.25 kHz, NEX = 1, slices = 192.

### Image processing

**Fig. 1:**
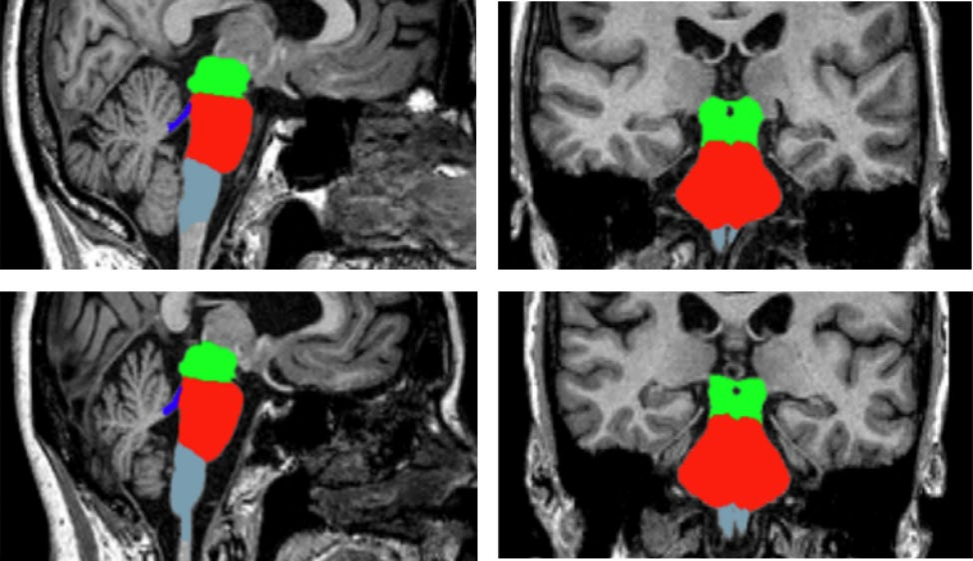
Automated brainstem parcellation. T1 brain MRIs were automatically parcellated by the brainstem procedure of FreeSurfer. The green label represents midbrain, red is pons, blue is superior cerebellar peduncle, and light bule is medulla oblongata

The volumes of 4 brainstem substructures, including, midbrain, pons, medulla oblongata, and superior cerebellar peduncle (SCP), and the whole brainstem were calculated from T1 images from each participant by using the automated procedure for volumetric measures in the FreeSurfer version 6.0 (Massachusetts General Hospital, Boston, U.S., http://surfer.nmr.mgh.harvard.edu). The subregional brainstem segmentation technique, developed by JE Iglesias, K Van Leemput, P Bhatt, C Casillas, S Dutt, N Schuff, D Truran-Sacrey, A Boxer and B Fischl [36] was applied to estimate the volumes of 4 brainstem substructures for all participants. This method allows for brainstem substructure segmentation using a robust and accurate Bayesian algorithm and has been specifically described in a previous publication [36]. In brief, the preprocessing procedures included skull stripping, bias field correction [37], automated Talairach transformation to the standard space of each subject’s brain [38, 39], intensity normalization, and the segmentation of subcortical structures [38]. After the above-mentioned procedures were performed, the preprocessed brain images were fed into the fully automatic segmentation algorithm implemented in the FreeSurfer and the volumes of the midbrain, pons, medulla oblongata, and SCP, and the entire brainstem were calculated. The automated parcellation of brainstem regions in our analysis is shown in Fig. 1.

### Statistical analyses

All statistical analyses were performed using Statistical Package for the Social Sciences (SPSS) version 27.0 (IBM Corp., Armonk, NY, USA). For categorical and continuous variables, the Chi-square tests (*χ*^*2*^) and analyses of variance (ANOVAs) were used for statistical analyses respectively. The volumes of the whole brainstem and its substructures in three groups were tested using ANOVAs and LSD method was used for post-hoc between-group comparison analyses. All volumetric analysis statistics were corrected for multiple comparison problems using the false discovery rate (FDR) method by Benjamini and Hochberg (BH). Finally, the association analyses between brainstem volumes and clinical features in all MDD patients were further performed with age, sex and educational level as covariates. The level of two-tailed statistical significance was set at *p* < 0.05 for all tests.

## Results

### Demographic and clinical characteristics

Demographic and clinical features of MDD patients and HCs in the sample are listed in Table 1. No significant difference was found in age (*F* = 0.033, *p* = 0.967), sex (*χ*2 = 1.576, *p* = 0.455), and educational level (*F* = 1.122, *p* = 0.329) among three groups of individuals. The two groups of MDD patients did not differ with respect to onset age (*F* = 3.251, *p* = 0.076) and scores of HAMD-17 (*F* = 0.021, *p* = 0.886). As we would expect, the RMDD group showed significantly prolonged illness duration (*F* = 34.20, *p* < 0.01) and increased times of depressive episodes.

**Table 1:** Demographic and clinical characteristics for all subjects (n = 111)

	FMDD n = 36 means(SD)	RMDD n = 25 means(SD)	HCs n = 50 means(SD)	Analysis *F/χ* ^*2*^	*p*-values
Age	29.1(7.68)	28.8(6.81)	29.3(8.56)	0.033	0.967
Gender (Male/Female)	11/25	6/19	19/31	1.576	0.455
Education years	14.0(3.34)	14.3(2.54)	14.9(2.51)	1.122	0.329
Illness duration (months)	10.33(10.81)	46.6(32.9)	/	34.20	< 0.01
Number of episodes	/	2.64(0.86)	/	/	/
Onset age	28.5(7.84)	24.9(7.31)	/	3.251	0.076
HAMD	24.9(3.70)	25.0(3.72)	/	0.021	0.886

FMDD, First-episode Major Depressive Disorder; HAMD, Hamilton Depression Scale; HCs, Healthy Controls; RMDD, Recurrent Major Depressive Disorder; SD, Standard Deviation

### Brainstem volume differences among groups

**Fig. 2:**
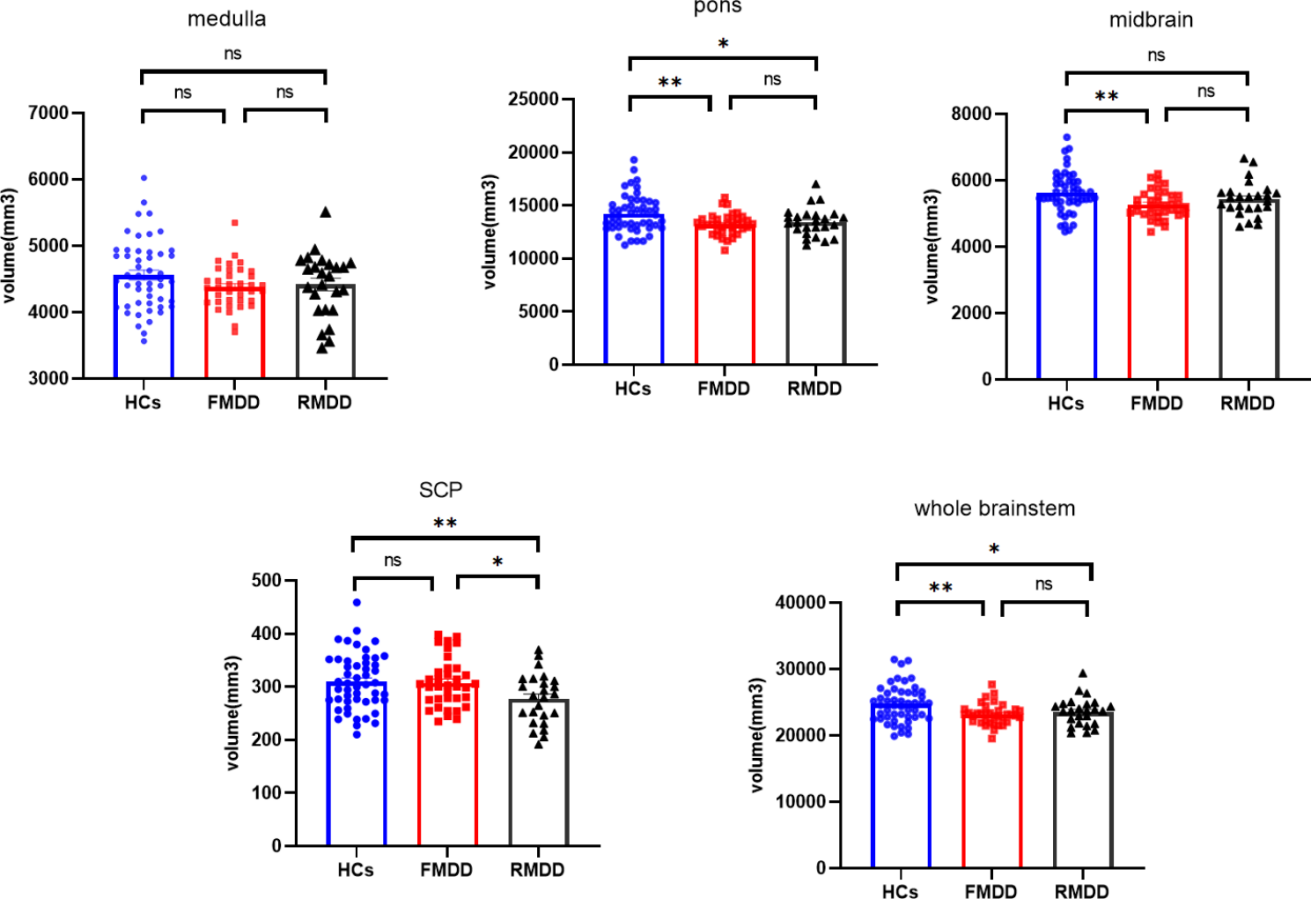
Comparisons of volumes of brainstem regions among three groups. FMDD, First-episode Major Depressive Disorder; HCs, Healthy Controls; RMDD, Recurrent Major Depressive Disorder; SCP, Superior Cerebellar Peduncle. *p < 0.05, **p < 0.01

The ANOVA revealed significant brainstem volumetric differences among three groups in the pons, midbrain, SCP, and the whole brainstem (*F* = 3.996 ~ 5.886, adjusted *p* = 0.015 ~ 0.028). As compared with HCs, both groups of MDD patients showed decreased volumes in the pons as well as the entire brainstem (*t* = 2.151 ~ 3.196, *p* = 0.002 ~ 0.034), however, only the FMDD group demonstrated a significantly reduced volume in the midbrain (*t* = 2.996, *p* = 0.003). Specifically, the RMDD group exhibited significantly decreased SCP volume when comparing to both FMDD (*t* = 2.347, *p* = 0.021) group and HCs (*t* = 2.685, *p* = 0.008). The detailed information for brainstem volumes of each group is summarized in Table 2; Fig. 2.

**Table 2:** Differences of volumes of brainstem regions among three groups

Brainstem regions	FMDD vs. RMDD vs. HCs								
	FMDD (n = 36)	RMDD (n = 25)	HCs (n = 50)	FMDD vs. RMDD vs. HCs			FMDD vs. HCs	RMDD vs. HCs	FMDD vs. RMDD
				*F*	*p*	adjusted *p*	*p*	*p*	*p*
Medulla oblongata	4377.79± 317.10	4417.05± 482.00	4559.47± 531.60	1.827	0.166	0.166	0.074	0.210	0.744
Pons	13228.29± 1008.35	13407.05± 1335.38	14246.53± 1755.68	5.886	**0.004**	**0.020**	**0.002**	**0.021**	0.639
SCP	307.19± 46.08	276.87± 47.84	309.51± 52.82	3.996	**0.022**	**0.028**	0.831	**0.008**	**0.021**
Midbrain	5274.79± 417.13	5431.14± 520.15	5626.28± 614.90	4.566	**0.012**	**0.020**	**0.003**	0.141	0.266
Whole brainstem	23188.05± 1579.86	23532.11± 2115.81	24741.79± 2764.38	5.368	**0.006**	**0.015**	**0.002**	**0.034**	0.566

Data are mean ± standard deviation (mm^3^)

The adjusted *p* values were obtained using Benjamini and Hochberg (BH) correction

FMDD, First-episode Major Depressive Disorder; HCs, Healthy Controls; RMDD, Recurrent Major Depressive Disorder; SCP, Superior Cerebellar Peduncle

Finally, correlation analyses revealed that the SCP volumes were negatively associated with the number of depressive episodes (*r*=-0.36, *p* < 0.01) and illness duration (*r*=-0.28, *p* = 0.035) in patients with MDD. In addition, we found that the SCP volumes were still negatively associated with the number of depressive episodes (adjusted *p* = 0.025) after BH correction.

## Discussion

In this study, regional volumetric alterations of the brainstem were investigated in patients with FMDD and RMDD. The current findings revealed that decreased regional and entire volumes of the brainstem were involved in the pathophysiology of MDD, and the volume of SCP might be specifically influenced by disease duration of recurring episodes of depression.

The brainstem, a crucial relay for motor and sensory pathways, also plays a role in regulating various physiological processes, such as cardiorespiratory control, arousal, and sleep [40]. The midbrain is a key area implicated in the brainstem and is believed to be associated with the development of MDD [16]. The pons is adjacent to the midbrain, which may be functionally linked to the midbrain. In the midbrain and pons, several nuclei, such as the VTA, DRN, and locus coeruleus, are associated with important monoaminergic neurotransmitters (dopamine, serotonin, and norepinephrine) in MDD [22, 41]. Our study found that depressive individuals exhibited decreased volumes of the pons, midbrain, as well as the entire brainstem when comparing to HCs, which might reflect altered functioning of the monoaminergic transmitters in MDD.

The current study is in parallel with previous research findings which showed significant structural alterations in the brainstem regions in MDD patients compared to HCs [28, 42, 43]. Consistently, a study in geriatric depression evaluating deep brain structures with transcranial sonography (TCS) found that reduced echogenicity (interrupted/invisible echogenic line) of brainstem raphe was significantly higher in the depressed group [44]. For sub-region studies, it has been reported that adults diagnosed with MDD showed significantly smaller volumes in the midbrain, relative to non-depressed adults [16, 45]. In a recent study, maternal antenatal depression was elucidated to be associated with decreased volumes in infant midbrain in early postnatal life, and that this was not accounted for by medication exposure [46]. Moreover, in patients with acute ischemic stroke, experiencing brainstem and deep cerebral microbleeds were more likely to be associated with the development of post-stroke depression (PSD) [47]. Taken together, our findings extend existing research to suggest that alterations in brainstem anatomy may play an important role in the pathophysiology of MDD.

Another noteworthy finding of this study was smaller SCP volumes of patients with RMDD compared to both FMDD and HCs. The SCP is a crucial pathway connecting the cerebellar cortex, especially the posterior cerebellar lobes, the deep cerebellar nuclei and the whole brain [48]. It is a critical structure in the prefrontal-thalamic-cerebellar circuit and disconnection in this brain network is thought to be associated with both cognitive and affective functions [49, 50]. Additionally, the SCP may have direct and indirect connections to the vagus nerve through the parabrachial nucleus that surrounds it, and thus has direct and indirect connections to depression-related cortical-limbic-thalamic-striatal neural circuits, which are key brain regions for RMDD [51, 52]. In this context, prior studies have already demonstrated the relationship between SCP and depressive symptoms in both MDD and bipolar disorder-II (BD-II) patients [31, 53].

Our study further revealed that decreased volumes of the SCP in MDD might be a biological marker of depressive relapse specifically. The current finding was supported by a previous diffusional kurtosis imaging (DKI) study in patients with MDD and BD. It was found that patients with MDD, but not BD exhibited significant differences from controls for DKI measures and cerebral blood flow (CBF) in bilateral SCP, suggesting that microstructural abnormality in the SCP might be a key neurobiological feature of MDD. Interestingly, correlation analysis showed there were associations between illness duration and DKI measures in the right SCP in MDD [54]. These findings might provide indirect evidence to confirm the relationship between SCP abnormalities and depressive relapse since long illness duration usually represented recurrence. Moreover, we also found significant negative associations of SCP volumes with illness duration and number of depressive episodes in MDD patients, which further suggested that volumetric reduction in the SCP might be a neurobiological marker for RMDD.

Several limitations of our study should be mentioned. First, the subregional brainstem segmentation technique developed by JE Iglesias, K Van Leemput, P Bhatt, C Casillas, S Dutt, N Schuff, D Truran-Sacrey, A Boxer and B Fischl [36] was the only method used in the present study, it will be better to combine multiple modalities in brainstem subregions to capture more information related to MDD in future studies. Second, we were unable to elucidate the precise structural changes in various subregions associated with the monoaminergic system, such as the DRN, VTA, or locus coeruleus. We also could not distinguish alterations of gray or white matter volumes in our analysis. Thus, the exact origins of the volume changes in the brainstem could not be identified. Third, previous studies have shown that the use of antidepressants may affect brainstem volume. Lai et al. observed that duloxetine, a kind of serotonin and norepinephrine reuptake inhibitor, possibly contributed to modest increases in the volumes of the brainstem, nucleus accumbens, putamen and hippocampus in MDD patients [55]. Meanwhile, Han et al. reported that greater brainstem volumes in drug-naïve MDD patients and found that the midbrain volume returns to normal after antidepressant treatment, and is even reduced, when the patient is in remission, which suggested the possible associations between midbrain volume changes and antidepressant medication in patients with MDD [30]. Therefore, although we recruited the patients with drug-naïve patients with first-episode depression or recurrent depression with continued withdrawal of more than 3 months, the changes of midbrain volumes in RMDD patients may be associated with previous use of anti-depression medication. Moreover, this study was a cross-sectional design, the causal relationship of altered brainstem volumes with MDD could not be directly determined.

## Conclusion

The present findings provided evidence of decreased brainstem volume involving in the pathophysiology of MDD, particularly, volumetric reduction in the SCP might represent a neurobiological marker for RMDD. Further research is needed to confirm our observations and deepen our understanding of the neural mechanisms underlying depression recurrence.

## Data Availability

The datasets generated and/or analyzed during the current study are not publicly available due to privacy and ethical restrictions but are available from the corresponding author on reasonable request.
